# Evaluation of long‐term chronic pain and outcomes for unilateral vs bilateral circular incision transabdominal preperitoneal inguinal hernia repair

**DOI:** 10.1002/ags3.12556

**Published:** 2022-02-16

**Authors:** Shunsuke Hayakawa, Tetsushi Hayakawa, Kaori Watanabe, Kenta Saito, Hirotaka Miyai, Ryo Ogawa, Minoru Yamamoto, Kenji Kobayashi, Shuji Takiguchi, Moritsugu Tanaka

**Affiliations:** ^1^ Department of General surgery Kariya Toyota General Hospital Kariya Japan; ^2^ Department of Gastroenterological Surgery Nagoya City University Graduate School of Medical Sciences Nagoya Japan; ^3^ Department of Laparoscopic Hernia Surgery Center Kariya Toyota General Hospital Kariya Japan

**Keywords:** chronic pain, inguinal hernia, laparoscopic surgery, propensity repair, recurrence

## Abstract

**Aim:**

This study has two aims: to evaluate long‐term chronic pain and complications after circular incision transabdominal preperitoneal inguinal hernia repair (C‐TAPP) and compare outcomes of unilateral and bilateral inguinal hernia cases.

**Methods:**

A postoperative patient questionnaire was used to evaluate pain and complications in 1546 patients who underwent C‐TAPP for simple inguinal hernia. Questions concerned satisfaction with surgery, pain at rest, pain at movement, mesh discomfort on a 10‐point scale, and complications, such as recurrence. Patients were classified into unilateral (U Group) and bilateral (B Group) groups, and propensity score matching was performed to compare long‐term chronic pain and complications.

**Results:**

The questionnaire return rates were 77.5% (1034 cases) and 79.9% (135 cases) in unilateral and bilateral cases. The frequency of moderate‐to‐severe (≥4 points) pain at rest, pain at movement, and mesh discomfort were 3.2%, 3.6%, and 4.5%, respectively. After propensity score matching, no significant differences in pain at rest (*P* = .726), at movement (*P* = .712), or mesh discomfort (*P* = .981) were detected between the U and B groups. Postoperative complications occurred in 2.1% of all patients, and the recurrence rate was 0.3%. In the post‐match comparison, no differences in complications with Clavian‐Dindo classification ≥III (U Group 0.7%, B Group 2.1%, *P* = .622) were detected.

**Conclusion:**

C‐TAPP, which focuses on the layered structure, showed acceptable results for long‐term chronic pain. Bilateral cases did not have worse pain or complications compared to unilateral cases.

## Graphical abstract

Circular incision TAPP, which focuses on the layered structure, showed acceptable results for long‐term chronic pain. Bilateral cases did not have worse pain or complications compared to unilateral cases.

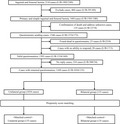

## 1 INTRODUCTION

Transabdominal preperitoneal inguinal hernia repair (TAPP) was first reported by Arregui, and case series were first reported in 1990.^1^, ^2^ TAPP is now a common surgical technique for inguinal hernia repair. However, chronic pain in inguinal hernia surgery is a major complication that affects the long‐term prognosis of patients. Thus, evaluating the incidence of chronic pain after TAPP is important. In recent studies, complication and recurrence rates were similar to the anterior approach, but postoperative pain was reduced.^3^, ^4^, ^5^, ^6^ Laparoscopic surgery reportedly incurs less chronic pain than the anterior approach.^5^, ^7^, ^8^, ^9^ However, according to the world guidelines, insufficient evidence supports TAPP for decreasing chronic pain due to the lack of clear endpoints and the dependence on surgeons' skills. Therefore, further investigation is needed.^10^

Globally, the peritoneal incision is made from the head side of the hernia and the peritoneum is dissected to the dorsal side for parietalization during TAPP.^2^, ^11^ In Japan, however, a surgical technique called circular incision TAPP (C‐TAPP) is more common. In C‐TAPP, a peritoneal incision is made from the dorsal side of the hernia internal ring, and a circular incision is made around the processus vaginalis, recognizing the layer structure, to proceed with parietalization. In C‐TAPP, a mesh is inserted in the layer close to the abdominal cavity to reduce pain. No studies report the postoperative outcomes, including chronic pain, in more than 1000 patients who underwent C‐TAPP.

One advantage of TAPP is that it does not increase the number of wounds compared to unilateral cases, even if bilateral surgery is performed. We often experience the incidental finding of an asymptomatic inguinal hernia on the contralateral side during surgery. In such cases, there is no clear conclusion about whether both sides should be repaired simultaneously. Whether there is a difference in complications and pain between bilateral and unilateral cases affects the decision of the initial surgical plan. However, only a few studies have examined the impact of bilateral cases on postoperative complications and pain compared with unilateral cases.^12^ If we do not perform simultaneous bilateral repair, it is not uncommon that after a unilateral operation, another operation is needed for inguinal hernia on the contralateral side. We thought that it was necessary to clarify the long‐term chronic pain and complications in bilateral cases.

In this study, we conducted a postoperative questionnaire survey to evaluate the surgical results of C‐TAPP, in terms of chronic pain and recurrence, and compared unilateral and bilateral cases.

Unlike cancer surgery, few institutions conduct long‐term surveillance for inguinal hernia repair; thus, visiting the hospital for follow‐up is difficult. Postoperative questionnaires are a common method to investigate the long‐term prognosis when patients do not visit the hospital for follow‐up.^3^, ^13^, ^14^, ^15^, ^16^, ^17^ In this study, the primary endpoints were the frequency and degree of chronic pain, and the secondary endpoints were other postoperative complications, such as recurrence.

## 2 METHODS

### 2.1 Patients

We performed 2141 inguinal hernia repair procedures (1892 unilateral and 249 bilateral) in inguinal hernia patients aged 16 years or older at our institution from April 2006 to March 2014. Patients undergoing emergency surgery, anterior approach, post‐prostatectomy, recurrent surgery, and simultaneous other surgeries were excluded (total 460 cases). As a result, 1681 patients (1501 unilateral cases and 180 bilateral cases) who underwent TAPP for simple inguinal hernia were included in the study. A postoperative inguinal hernia questionnaire was used to collect data. Unilateral cases were defined as the Unilateral Group (U Group), and bilateral cases were defined as the Bilateral Group (B Group). The protocol for this research project has been approved by a suitably constituted Ethics Committee of the institution and it conforms to the provisions of the Declaration of Helsinki (Ethics Review Committee of Kariya Toyota General Hospital, Approval No. 205). As this is a retrospective study, patient consent was obtained on an opt‐out basis.

### 2.2 Surgical indication and technique

**FIGURE 1:**
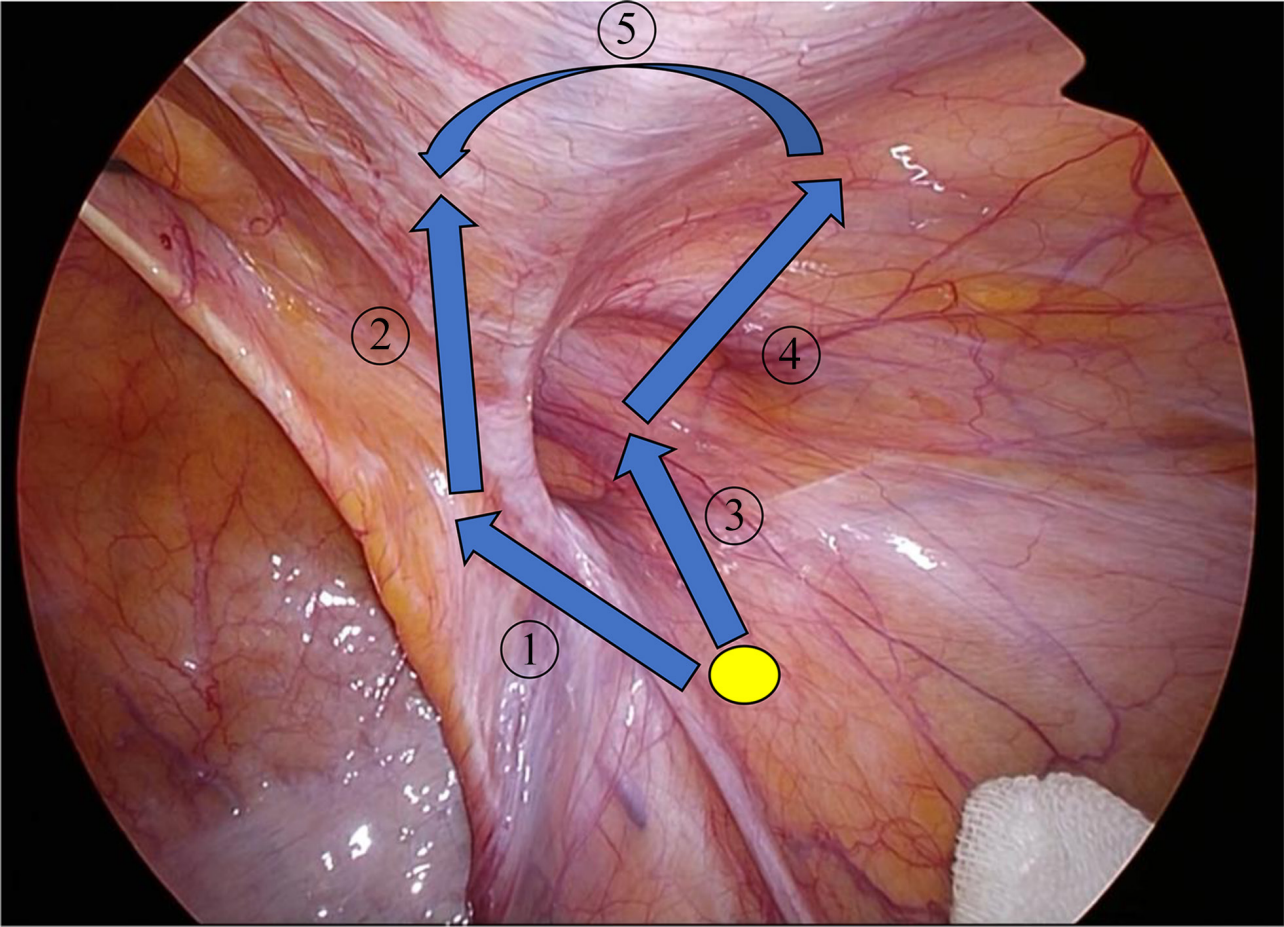
Peritoneal incision procedure in circular incision transabdominal preperitoneal inguinal hernia repair (C‐TAPP) (right side case). The incision is made by following the blue arrow from the yellow circle on the dorsal side

**FIGURE 2:**
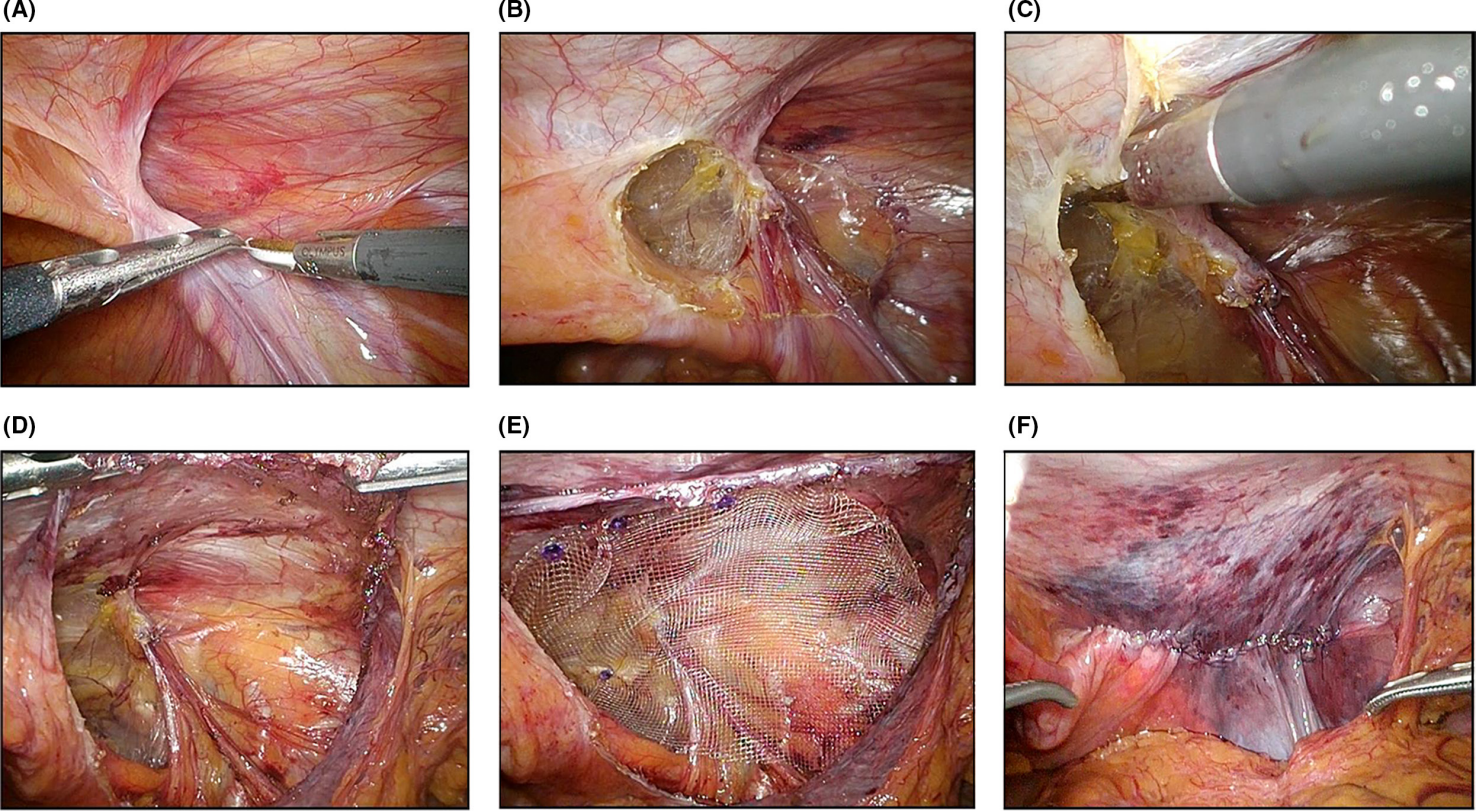
Intraoperative photos showing the surgical procedure. (A) The peritoneal incision was made on the dorsal side of the hernia orifice near the outer side the spermatic vessels. (B) The preperitoneal cavity was entered from the medial side of the ductus deferens while preserving the vas deferens and spermatic veins on the abdominal wall. (C) The incision was made at the midline of the inferior epigastric vessels and was intersected with a line from the dorsal side. (D) A space for mesh was created on the myopectineal orifice. (E) Mesh expansion was performed. (F) The peritoneum was closed with continuous sutures

We used a circular incision around the peritoneal processus vaginalis (Figure 1). The peritoneal incision was made on the dorsal side of the hernia orifice near the outer side the spermatic vessels (Figure 2A). This incision was made to enter the layer between the peritoneum and the preperitoneal fascia areolar layer on the dorsal side of the hernia orifice. While maintaining this layer, tissue was dissected toward the medial side. The preperitoneal cavity was entered from the medial side of the ductus deferens while preserving the vas deferens and spermatic veins on the abdominal wall (Figure 2B). A lateral to medial peritoneal incision was made at the superior side of the hernia orifice. The incision was made at the midline of the inferior epigastric vessels and was intersected with a line from the dorsal side (Figure 2C). On the head side, the attenuated posterior rectus sheath (APRS) was preserved during the peeling process. On the dorsal side, parietalization was performed to preserve as wide an area of preperitoneal fascia areolar layer as possible. A space for mesh was created on the myopectineal orifice (Figure 2D) and mesh expansion was performed (Figure 2E). The type of mesh changed depending on the time of year, but the mesh was always placed in the same layer. The peritoneum was closed with continuous sutures (Figure 2F). The anatomical names of the layers in this section are in accordance with Fowler's report.^18^ The operators ranged from experienced surgeons with over 2,000 cases to residents. However, surgeons with more than 100 surgeries acted as either supervisors or operators in all surgeries for this study.

### 2.3 Location of mesh

**FIGURE 3:**
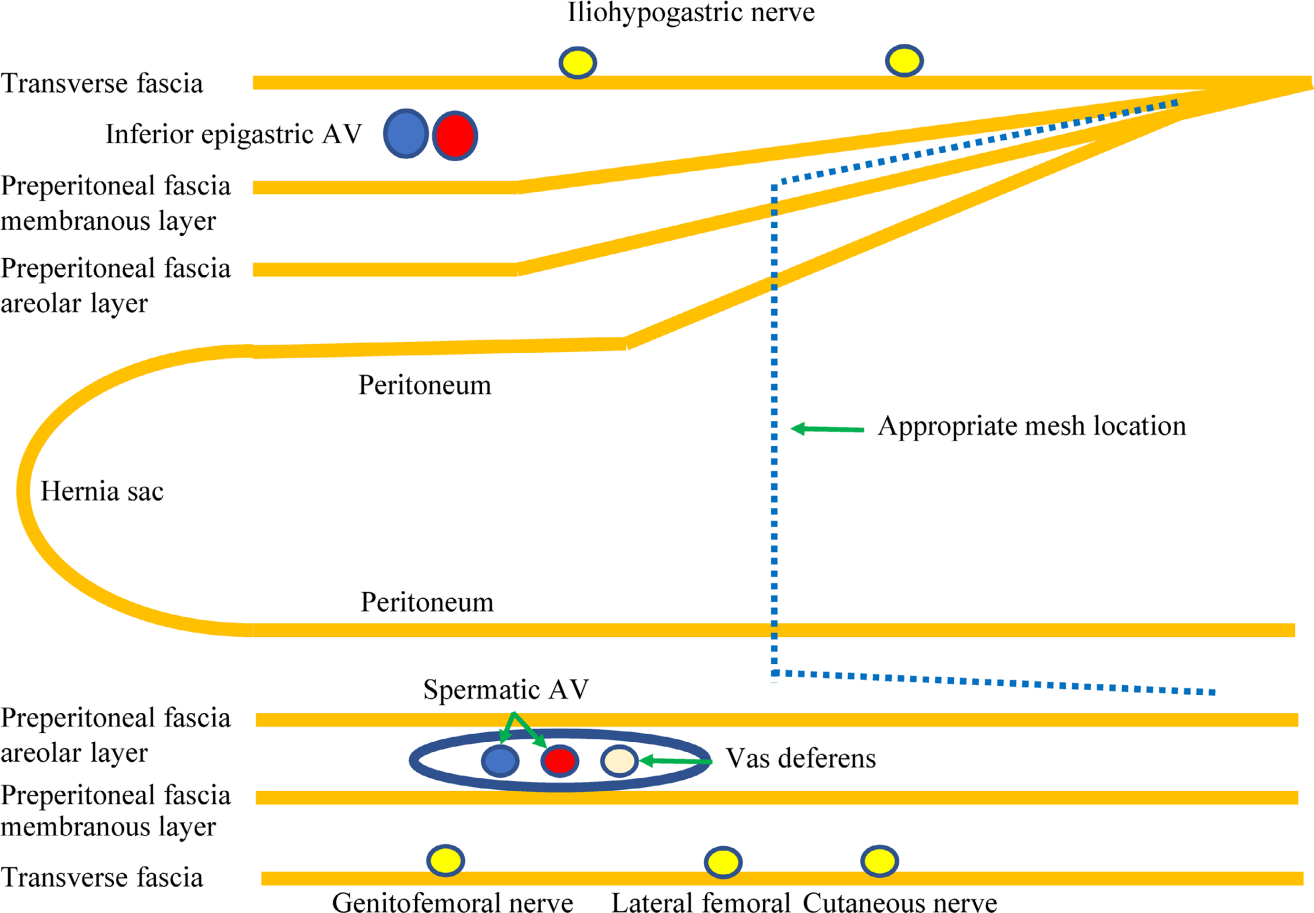
Schematic diagram showing the appropriate mesh location. Abbreviations: AV, artery and vein

The mesh was placed in a layer as far away from the nerves as possible. On the dorsal side, the mesh was placed between the preperitoneal fascia areolar layer and the peritoneum. On the head side, the mesh was placed between the APRS and the preperitoneal fascia areolar layer (Figure 3).

### 2.4 Regular follow‐up

Blood tests were performed before and the day after the surgery. Basically, the patient was discharged from the hospital the day after surgery. At the time of discharge, 10 doses of oral NSAIDs were prescribed for the patient. If no abnormalities occurred, the follow‐up period was set at 3 months postoperatively, and the follow‐up period was extended for symptomatic patients. The patients were advised to visit our outpatient clinic if any symptoms occurred after the follow‐up.

### 2.5 Questionnaire contents

**FIGURE 4:**
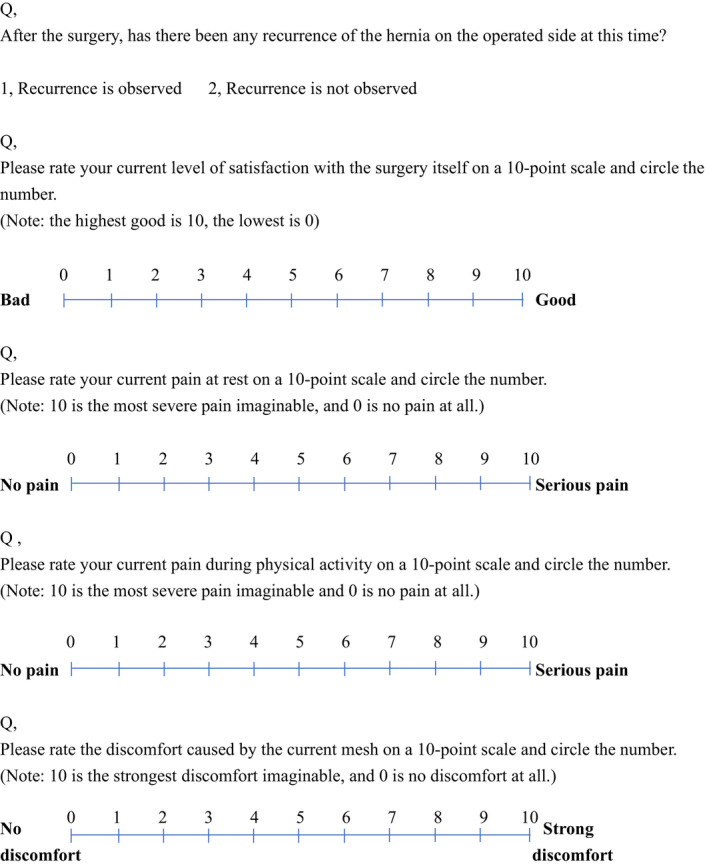
Questionnaire contents (excerpt from questionnaire sheet)

The presence or absence of recurrence after the follow‐up period was included in the questionnaire. Questions about surgical satisfaction (0‐10 point scale with 0 being the lowest and 10 being the highest), pain at rest and pain at movement according to the Numeric Rating Scale (NRS) (0‐10 point scale with 0 being no pain and 10 being the strongest pain), and mesh discomfort (0‐10 point scale with 0 being no discomfort and 10 being the strongest) were also included in the questionnaire (Figure 4).^19^ The degree of pain was classified according to the report by Fujita et al.^20^ Moderate or higher (4 points or higher) pain was defined as a symptom. The patients with a symptom were also asked whether they were visiting a hospital or not.

### 2.6 Questionnaire methods

The first questionnaire was sent by mail 1 year after the end of the study period, excluding patients whose deaths were confirmed in the hospital's medical records. For those cases that did not reply, the questionnaire was resent after 1 year and 11 months. For those cases that did not return the second questionnaire, we requested contact by phone. Patients with recurrence (on the questionnaire) were checked by telephone and examined in the outpatient clinic if necessary.

### 2.7 Statistical analysis

Statistical analyses were performed using the statistical software, EZR.^21^ Propensity score matching was performed to correct for confounding factors and background factors were adjusted. In the propensity score matching, one‐to‐one matching between the groups was performed using the nearest neighbor matching method with a caliper width of 0.2. Fisher's exact tests were used to compare categorical data. Mann‐Whitney *U* tests were used for continuous variables. Wilcoxon rank‐sum tests were used for pain level and satisfaction in the questionnaire. A significant difference was defined as *P* < .05.

## 3 RESULTS

### 3.1 Questionnaire response

**FIGURE 5:**
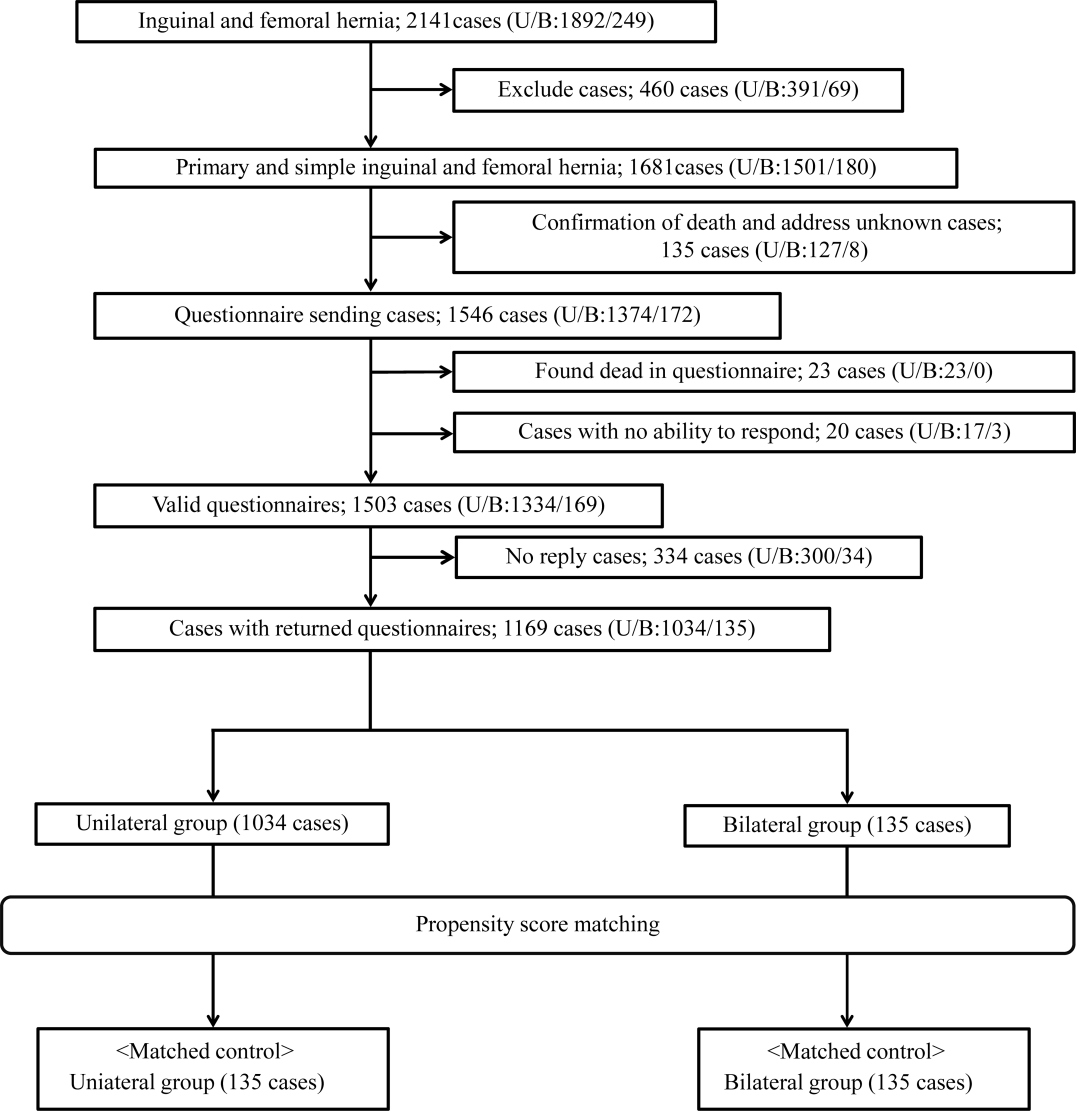
Flowchart of TAPP patients who participated in this questionnaire survey. Abbreviations: B, bilateral cases; TAPP, Transabdominal preperitoneal inguinal hernia repair; U, unilateral cases

The number of cases in the questionnaire study is shown in the flowchart (Figure 5). A total of 1681 patients were selected, excluding patients with unknown addresses, deceased patients, and patients with dementia; 1503 valid questionnaires were obtained, excluding patients who were dead and those whose questionnaires returned unanswered. Of these, 1169 were returned. The response rate was 77.8%. The median postoperative duration at the time of the survey was 50.7 (13‐119) months in the returned cases.

### 3.2 Unilateral cases

Among the 1501 unilateral cases selected, 45 deaths were confirmed by medical records, and 1456 questionnaires were prepared. However, the addresses on 82 of these were invalid, leaving 1374 questionnaires that were mailed. Twenty‐three of the surveyed patients were confirmed to be deceased by the questionnaire, and 17 patients were unable to answer due to dementia. Of the remaining questionnaires, 1034 were returned and 300 cases were not returned. The response rate was 77.5%.

### 3.3 Bilateral cases

Among the 180 cases selected, no deaths were confirmed by our medical records and eight patients had unknown addresses. Therefore, 172 questionnaires were sent out. Three patients were unable to answer the questionnaire due to dementia. Of the remaining questionnaires, 135 were returned and 34 were not returned. The response rate was 79.9%.

### 3.4 Patient backgrounds

Patient backgrounds before and after propensity score matching were categorized into Unilateral Cases (U Group) and Bilateral Cases (B Group), as shown in Table 1. Before propensity score matching, the B Group was significantly older and had lower body mass index and worse American Society of Anesthesiologists scores (ASA). After propensity score matching, significant differences in each parameter were adjusted. The type of inguinal hernia and the size of the hernia orifice are described in Table 1.

**TABLE 1:** Patient backgrounds and type of hernia

Back grounds	All patients				Propensity matching patients			
	U group (1034)	B group (135)		*P*	U group (135)	B group (135)		*P*
Age, years^a^	63 (55,71)	69 (62,74)		<.001	68 (61,76)	69 (62,74)		.874
Gender (male)	920 (89.0)	122 (90.4)		.768	117 (86.7)	122 (90.4)		.446
BMI, kg/m^2^ ^a^	22.8 (21.0, 24.4)	21.9 (20.4, 23.6 )		<.001	21.4 (20.2, 23.6)	21.9 (20.4, 23.6)		.367
ASA Score (1/2/3/4/5)	423/605/6/0/0	28/105/2/0/0		<.001	31/101/3/0/0	28/105/2/0/0		.786
Abdominal surgical history	343 (33.1)	52 (38.5)		.246	45 (33.3)	52 (38.5)		.447
Antithrombotic therapy	109 (10.5)	15 (11.1)		.882	14 (10.4)	15 (11.1)		1.0
Side of hernia defect (Right)	566 (54.7)	—		—	86 (63.7)	—		—
**Hernia type**		**Right**	**Left**			**Right**	**Left**	
Indirect	774 (74.9)	50 (37.0)	54 (40.0)	—	91 (67.4)	50 (37.0)	54 (40.0)	—
Direct	198 (19.1)	66 (48.9)	66 (48.9)	—	36 (26.7)	66 (48.9)	66 (48.9)	—
Femoral	21 (2.0)	5 (3.7)	5 (3.9)	—	5 (3.7)	5 (3.7)	5 (3.9)	—
Combined	39 (3.8)	14 (10.4)	9 (6.7)	—	3 (2.2)	14 (10.4)	9 (6.7)	—
Others	2 (0.2)	1 (0.7)	1 (0.7)	—	0	1 (0.7)	1 (0.7)	—
Size of defect		Right	Left	—		Right	Left	—
<3.0	665 (64.3)	75 (55.6)	74 (54.9)	—	83 (61.5)	75 (55.6)	74 (54.9)	—
>3.0	356 (35.7)	60 (44.4)	60 (44.4)	—	52 (38.5)	60 (44.4)	60 (44.4)	—
Unidentified	13 (1.3)	0	1 (0.7)	—	0	0	1 (0.7)	—

Note

Data are shown as number of patients (%).

Abbreviations: ASA, American Society of Anesthesiologists; BMI, body mass index.

^a^ median, IQR interquartile range (25%, 75%).

### 3.5 Surgical outcome and blood test results

Surgical outcome and blood test results are described in Table 2. Before and after propensity score matching, white blood cell count, and C‐reactive protein on postoperative day 1 were higher, and the operation time was prolonged in Group B compared with these parameters in Group U (*P* < .001). No significant differences in the amount of bleeding, postoperative hospital stay, the proportion able to be discharged on the day after surgery, intraoperative complications, postoperative complications, and frequency of complications (Clavian‐Dindo classification ≥III) were detected between the two groups (Table 2).

**TABLE 2:** Surgical outcome and complications

Surgical outcome and blood sampling results	All patients			Propensity matching patients		
	U group (1034)	B group (135)	*P*	U group (135)	B group (135)	*P*
Preoperative WBC (/μL)^c^	5700 (4900‐6800)	5800 (5100‐6700)	.632	5800 (4900‐6950)	5800 (5100‐6700)	.804
POD1 WBC (/μL)^c^	7700 (6500‐8850)	7950 (6650‐9150)	.196	7700 (6700‐9150)	7950 (6650‐9150)	.464
POD1 CRP (mg/dL)^c^	0.78 (0.47‐1.27)	1.04 (0.69‐1.95)	<.001	0.80 (0.48‐1.42)	1.04 (0.69‐1.95)	<.001
Operative time (min)^c^	88 (70‐103)	132 (104‐158)	<.001	82 (69‐82)	132 (104‐158)	<.001
Amount of bleeding (mL)^c^	2 (1‐3)	2 (2‐4)	<.001	2 (1‐3)	2 (2‐4)	<.001
Postoperative hospital stay (days)^c^	1 (1‐1)	1 (1‐1)	.119	1 (1‐1)	1 (1‐1)	.739
Proportion able to be discharged on the day after surgery	1015 (98.2)	130 (96.3)	.185	131 (97.0)	130 (96.3)	1.0
Intraoperative complication	2 (0.2)	1 (0.7)	.308	2 (1.5)	1 (0.7)	1.0
Postoperative complications	21 (2.0)	4 (3.0)	.520	4 (3.0)	4 (3.0)	1.0
Clavian‐Dindo classification, >III	11 (1.1)	3 (2.2)	.213	1 (0.7)	3 (2.1)	.622
Clavian‐Dindo classification, (I/II/IIIa/IIIb/IV, V)	3/7/8/3/0	0/1/2/1/0	.494	0/3/0/1/0	0/1/2/1/0	.590
Intraoperative complication						
Small bowel injury	2 (0.2)	0	—	2 (1.5)	0	—
Remnants of a gauze fragment	0	1 (0.7)	—	0	1 (0.7)	—
Postoperative complications						
Surgical site infection	3 (0.3)	0	—	0	0	—
Seroma^a^	7 (0.7)	2 (1.5)	—	1 (0.7)	2 (1.5)	—
Additional analgesic medication	5 (0.5)	1 (0.7)	—	0	1 (0.7)	—
Mesh infection	0	0	—	0	0	—
Postoperative bleeding^b^	1 (0.1)	0	—	1 (0.7)	0	—
Recurrent	3 (0.3)	1 (0.7)	—	0	1 (0.7)	—
Adhesive intestinal obstruction	0	0	—	0	0	—
Others	2 (0.2)	0	—	2 (1.5)	0	—
Re‐operation	2 (0.2)	2 (1.5)	—	0	2 (1.5)	—

Note

Data are shown as number of patients (%).

Abbreviations: CRP, C‐reactive proteins; POD1, postoperative day 1; WBC, white blood cells.

^a^ Needed puncture.

^b^ More than Hb2g/dL decrease.

^c^ Median, IQR interquartile range (25%, 75%).

### 3.6 Details of complications

Complications are listed in Table 2. Two cases of small bowel injury as an intraoperative complication (due to surgical intervention) occurred. In both cases, only the serosa was damaged, and no perforation occurred. Therefore, the serous was sutured and the hernia was repaired using mesh as planned. In one case, a fiber fragment of gauze remained in the abdominal cavity and required reoperation for removal. There were nine cases (0.8%) of seroma requiring puncture or other treatment, including seven cases (0.7%) in the U Group and two cases (1.5%) in the B Group. Six patients (0.5%) required additional analgesic medication on an outpatient basis, including five patients (0.5%) in the U Group and one patient (0.7%) in the B Group. There were four recurrences, including three (0.3%) in the U Group and one (0.7%) in the B Group; one patient did not undergo reoperation. One patient (0.1%) with postoperative bleeding underwent interventional radiology. Reoperation was performed on four patients (0.3%), three (2 in Group U, 1 in Group B) were operated for recurrence and one was operated for removal of fiber fragments of gauze.

### 3.7 Questionnaire results

Questionnaire results are shown in Table 3. The frequencies of moderate‐to‐severe long‐term pain at rest, pain at movement, and mesh discomfort were 3.2%, 3.6%, and 4.5%, respectively, in all patients. No significant differences in the degree of satisfaction, pain at rest, pain at movement, and mesh discomfort between Group U and Group B before or after propensity score matching were detected. Among the patients with moderate or severe symptoms, 14 (30.0%) went to our hospital and only one (2.4%) went to another hospital.

**TABLE 3:** Questionnaire results (satisfaction and pain)

	All patients				Propensity matching patients		
	All cases (1169)	U group (1034)	B group (135)	*P*	U group (135)	B group (135)	*P*
Degree of satisfaction (Average)							
Very satisfied (9‐10)	963 (82.4)	848 (82.0)	115 (85.2)	.249	107 (79.3)	115 (85.2)	.133
Satisfied (7‐8)	135 (11.5)	122 (11.8)	13 (9.6)		20 (14.8)	13 (9.6)	
Normal (5‐6)	24 (2.1)	21 (2.0)	3 (2.2)		5 (3.7)	3 (2.2)	
Dissatisfied (3‐4)	11 (0.9)	11 (1.1)	0		0	0	
Very dissatisfied (0‐2)	6 (0.5)	6 (0.6)	0		0	0	
Blank	30 (2.6)	26 (2.5)	4 (3.0)		3 (2.2)	4 (3.0)	
Pain at rest (Average)							
No pain (0)	947 (81.0)	834 (80.7)	113 (83.7)	.288	113 (83.7)	113 (83.7)	.726
Mild pain (1‐3)	163 (13.9)	146 (14.2)	17 (12.6)		16 (11.9)	17 (12.6)	
Moderate pain (4‐6)	26 (2.2)	25 (2.4)	1 (0.7)		5 (3.7)	1 (0.7)	
Severe pain (7‐10)	12 (1.0)	11 (1.1)	1 (0.7)		0	1 (0.7)	
Blank	21 (1.8)	18 (1.7)	3 (2.2)		1 (0.7)	3 (2.2)	
Pain at movement (Average)							
No pain (0)	910 (77.8)	807 (78.0)	103 (76.2)	.801	109 (80.7)	103 (76.2)	.712
Mild pain (1‐3)	194 (16.6)	170 (16.4)	24 (17.8)		20 (14.8)	24 (17.8)	
Moderate pain (4‐6)	28 (2.4)	27 (2.6)	1 (0.7)		4 (3.0)	1 (0.7)	
Severe pain (7‐10)	14 (1.2)	13 (1.3)	1 (0.7)		0	1 (0.7)	
Blank	23 (2.0)	17 (1.6)	6 (4.4)		2 (1.5)	6 (4.4)	
Mesh discomfort (Average)							
No discomfort (0)	870 (74.4)	771 (74.6)	99 (73.3)	.326	105 (77.8)	99 (73.3)	.981
Mild discomfort (1‐3)	219 (18.7)	195 (18.9)	24 (17.8)		22 (16.3)	24 (17.8)	
Moderate discomfort (4‐6)	28 (2.4)	26 (2.5)	2 (1.5)		4 (3.0)	2 (1.5)	
Severe discomfort (7‐10)	24 (2.1)	24 (2.3)	0		1 (0.7)	0	
Blank	28 (2.4)	18 (1.7)	10 (7.4)		2 (1.5)	10 (7.4)	

Note

Data are shown as number of patients (%). The statistical analysis of this table was done in Wilcoxon rank‐sum test.

## 4 DISCUSSION

In this investigation of C‐TAPP cases, moderate‐to‐severe pain at rest was observed in 3.8% and pain at movement in 3.6%. Moderate‐to‐severe mesh discomfort was observed in 4.5%. No differences in any of the categories were detected between the U and B groups, even after propensity score matching. The results suggest that the two repair sites do not increase chronic pain if the layers are properly dissected by C‐TAPP.

According to the European Hernia Society guidelines, chronic pain after inguinal hernia repairs is associated with intraoperative nerve damage, and the incidence of chronic pain is lower with laparoscopic techniques, such as TAPP and the totally extra‐peritoneal approach (TEP).^22^ In a meta‐analysis conducted by Bullen et al, 12 studies were reviewed and laparoscopic surgery resulted in a lower risk of chronic pain compared to the anterior approach (considering the meta‐analysis design, various techniques were included).^23^ The incidence of chronic pain with laparoscopic surgery was 9.7% (168 out of 1780 patients) in the meta‐analysis; however, a mixture of TAPP and TEP techniques was included in the studies, and the definitions of chronic pain and the follow‐up period were not consistent.^23^ Takayama et al investigated the satisfaction level, frequency of pain, mesh discomfort, and numbness in laparoscopic surgery with the mesh plug and open tissue inguinal hernia repair; no differences in pain and mesh discomfort were detected, and the incidence of numbness was significantly lower in TAPP.^4^ However, as far as we know, the long‐term pain level after TAPP was not evaluated in a large number of patients (more than 1000) before our report.

Chronic pain of inguinal hernia is generally defined as pain that persists for at least 3 months after surgery.^10^ However, no specific diagnostic criteria for the evaluation of pain intensity exist, and the indicators vary among reports. Therefore, evaluation is difficult and sufficient evidence has not yet been collected. For tissue‐to‐tissue herniorrhaphy (Bassini, Mcvay, and Shouldice repairs), 62.9% of patients (moderate‐to‐severe pain: 11.9%) felt pain 1 year after the surgery, and 53.6% of patients (moderate‐to‐severe pain: 10.6%) felt pain 2 years after the surgery.^24^ In addition, a Danish hernia database report showed that 1 year after surgery, 28.7% of patients felt pain, 11% had difficulty with work or activities, and 4.5% required medical intervention.^25^ Although the different definitions make generalization difficult, the incidence of chronic pain in our report was lower than the incidence of chronic pain (9.7%) in the meta‐analysis by Bullen et al.^23^ In our surgical technique, the peritoneal incision is made from the dorsal side, the peritoneum and the preperitoneal fascia areolar layer are preserved in a wider area than in the conventional method, and the dissection and mesh are deployed without touching the vas deferens, spermatic vessels, and painful nervous system. We speculate that the differences in technique contribute to the reduced pain because the mesh is positioned in a wider area on the abdominal cavity side.

Interestingly, no differences in the rate of chronic pain were detected between groups U and B. We assumed that the rate of chronic pain would increase with repairs for two lesions, but no differences were detected. In TAPP, a contralateral inguinal hernia may be found incidentally at the time of surgery, even if the patient is asymptomatic. However, significantly higher postoperative complications and reoperation rates have been reported when repairing bilateral cases, which are disadvantages of simultaneous repair.^12^ Until now, each institution has had a different approach as to whether to repair bilateral cases simultaneously. Considering the results of this study, C‐TAPP, with its emphasis on membrane structure, may not increase the risk of chronic pain caused by inguinal hernia repair. Therefore, simultaneous bilateral repair was considered acceptable considering chronic pain and complications. The results suggest that simultaneous bilateral repair by C‐TAPP is a safe procedure with long‐term prognosis, although the long‐term safety of the procedure has not been conclusively determined. Complications other than chronic pain were also investigated as secondary outcomes in this study. Intraoperative complications occurred in three patients (0.3%), postoperative complications in 25 patients (2.1%), and recurrence in four patients (0.3%). According to various reports, the total complication rate of TAPP is 3.6%‐4.6%, and the recurrence rate is 0.6%‐1.2%.^4^, ^13^, ^26^, ^27^ The results of the present report of C‐TAPP are comparable to those of previous reports; thus, there is little risk of increased complications with this technique. This result may be a milestone in the performance of the C‐TAPP in comparison with other techniques.

The degree of a patient's preoperative symptoms may affect the postoperative symptoms. However, since this was an 8‐year retrospective study, quantitative data on preoperative symptoms were not collected. In addition, the number of analgesic medications, which is the most objective assessment of pain, could not be accurately measured because of the long‐term follow‐up of patients with chronic pain. To simplify the questionnaire, we did not ask questions about neuralgia and numbness. The questionnaire was designed to be as simple as possible, but there were a few missing answers, mainly from the elderly. Although we tried to confirm the results by phone calls and other means, few cases could not be confirmed. These points may be the limitations of this study. Another limitation of this study was that this was a single‐center study, although the number of cases was not small. In the future, a system to follow up patients on a large scale, such as the creation of a nationwide registry of patients, should be established and examined.

In conclusion, we performed C‐TAPP with emphasis on the layered structure. The results showed that the frequencies of moderate‐to‐severe long‐term pain at rest, pain at movement, and mesh discomfort were 3.2%, 3.6%, and 4.5%, respectively. Postoperative complications occurred in 2.1% of patients, and the recurrence rate was 0.3%. No differences in pain and complications were detected between the U and B groups before or after propensity score matching. The results of C‐TAPP are acceptable as a standard TAPP technique, and bilateral TAPP is not worse than unilateral TAPP in terms of pain and complications.

## DISCLOSURE

Conflict of Interest: Authors declare no conflict of interests for this article.

Informed Consent: As this is a retrospective study, patient consent was obtained on an opt‐out basis.

Approval of the Research Protocol: The protocol for this research project has been approved by a suitably constituted Ethics Committee of the institution and it conforms to the provisions of the Declaration of Helsinki. Ethics Review Committee of Kariya Toyota General Hospital, Approval No. 205.
